# 

**Published:** 1857-04

**Authors:** 


### SUBSCRIPTIONS RECEIVED.

Drs. S. S. Satchwell, N. C., 1st and 2d vol.; T. A. Boddie, Ga., 2d vol.; W. J. Hayes, Ala., 2d vol.; E. C. Hawes, Ga., 1st and 2d vol,; J. G. Broyles, Miss., 2d vol.; P. E. Jennings, Ga., 1st and 2d vol.; N. A. Moreland, Ga., 1st and 2d vol.; Wm. Matthews, Ga., 2d vol.; J. M. Darnall, Ga., 2d vol.; R. Search, Ala., 1st and 2d vol.; J. B. Fuller, Ala., 2d vol.; M. A. Leake, Ga., 2d vol.; Leonard Morgan, Ga., 1st and 2d vol.; Lewe Sessions, Ga., 1st and 2d vol.; Milton O. Stribling, Ga., 1st and 2d vol.; J. F. Woodbury, Ga., 2d vol.; E. C. Hood, Ga., 2d vol.; R. Mitchell, Tenn., 2d vol.

☞ All Communications pertaining to the Editorial Department of the Journal, should be addressed to the Editors.

☞ All Communications, having reference to the Subscription, the Advertising or the Financial Department, should be addressed to the Treasurer, J. G. Westmoreland, M. D., Dean of the Faculty of the Atlanta Medical College.

